# Effects of blood and root-dentin cleaning on the porosity and bond strength of a collagen bioceramic material

**DOI:** 10.1590/0103-6440202405907

**Published:** 2024-07-22

**Authors:** F. M. Saltareli, L. M. S. Castro-Raucci, C. E. S. Miranda, N. C Tavella-Silva, I. R. Oliveira, W. Raucci-Neto

**Affiliations:** 1. School of Dentistry, University of Ribeirão Preto, Ribeirão Preto, SP Brazil. Av Costábile Romano, 2201, Ribeirão Preto, SP, 14096-000, Brazil; 2. Institute for research and Development, University of Vale do Paraíba, São José dos Campos, SP, Brazil. Av. Shishima Hifumi, 2911, Urbanova, São José dos Campos - SP, 12244-390

**Keywords:** dentin, blood, calcium silicate, porosity, dental bonding, dentina, sangue, silicate de cálcio, porosidade, adesão dentaria

## Abstract

To assess the effect of cleaning protocols on dentin contaminated with blood in reparative endodontic materials, bovine root samples were divided: no contamination (N); contamination (P); contamination and cleaning with saline (S), 2.5% NaOCl+saline (Na) or 2.5% NaOCl+17% EDTA+saline (NaE) and filled with: mineral trioxide aggregate (MTA), calcium-aluminate-cement (C), or C+collagen (Ccol) (n=13). The samples were evaluated for porosity, chemical composition, and bond strength. MTA porosity was lower than C (p=0.02) and higher than Ccol (p<0.001). P and NaE were similar (p=1.00), but higher than the other groups (p<0.001). MTA bond strength was similar to Ccol (p=0.777) and lower than C (p=0.028). P presented lower bond strength than the N (p<0.001); S and Na were similar to each other (p=0.969), but higher than P and lower than N (p<0.001). It was observed a predominance of mixed and cohesive failures. None of the samples showed Ca/P ratio values similar to human hydroxyapatite. This study showed that contamination with blood increased the materials porosity, but dentin cleaning with 2.5% NaOCl reduced this effect, and the collagen additive reduced the material porosity. Furthermore, blood contamination reduced the materials bond strength, and cleaning with saline or 2.5% NaOCl diminished this effect.

## Resumo

As amostras de raízes bovinas foram divididas em: sem contaminação (N); contaminação sanguínea (P); contaminação sanguínea e limpeza com soro fisiológico (S), 2,5% NaOCl+soro fisiológico (Na) ou 2,5% NaOCl+17%EDTA+soro fisiológico (NaE) e preenchido com: agregado de trióxido mineral (MTA), cimento de aluminato de cálcio (C), ou C+colágeno (Ccol) (n=13). A porosidade do MTA foi menor que C (p=0,02) e maior que Ccol (p<0,001). P e NaE foram semelhantes (p=1,00), mas superiores aos demais grupos (p<0,001). A resistência de união do MTA foi semelhante ao Ccol (p=0,777) e inferior ao C (p=0,028). P apresentou menor resistência de união que o N (p<0,001); S e Na foram semelhantes entre si (p=0,969), porém maiores que P e menores que N (p<0,001). Este estudo mostrou que a contaminação com sangue aumentou a porosidade dos cimentos, mas a limpeza da dentina com NaOCl 2,5% reduziu esse efeito, e o aditivo de colágeno reduziu a porosidade dos cimentos. Além disso, a contaminação sanguínea reduziu a resistência de união dos cimentos e a limpeza com solução salina ou NaOCl 2,5% diminuiu esse efeito.

## Introduction

Surgical endodontic treatment may be indicated for various clinical situations, such as persistent or recurrent infections with persistent bone resorption, treatment of complex root canals, dental fractures, removal of cysts or tumors, repair of inaccessible root canals, preparation for dental prosthetics, or when an accident occurs during endodontic treatment ^1^. This technique involves preparing and sealing the root dentin with bioceramic-based materials that possess several properties, such as dimensional stability, radiopacity, antimicrobial activity, biocompatibility, and bioactivity ^2^.

Different formulations of calcium silicate cements and new materials, such as calcium aluminate cements, are being developed and evaluated to improve the clinical performance of surgical endodontic treatment ^2^ ^,^ ^3^. It has been demonstrate that calcium aluminate cements have similar physical-chemical properties and mechanical resistance under compressive loads compared to calcium silicate cements; additionally, it has demonstrated improved biological responses by supporting the acquisition of the osteogenic and odontogenic cell phenotype ^4^ ^,^ ^5^.

Among the new additives that can improve the properties of dentin repair materials, collagen plays an important role in maintaining the biological integrity and flexibility during tissue repair, owing to its ability to undergo constant remodeling to refine cellular behavior and function ^6^. Therefore, collagen is a promising additive that can improve the interaction between the material and the repair tissue ^7^.

Reparative endodontic materials are usually exposed to body fluids, such as blood, which could alter their properties and impair their ability to prevent the infiltration and proliferation of microorganisms, thus compromising the success of the surgical treatment ^8^. The greater the porosity of the material, the higher the risk of penetration of contaminated body fluids ^9^. The protein components of these fluids can affect the bonding of the repair material to the dentin, leading to inefficient sealing ^10^. Furthermore, blood can be incorporated during the initial setting reaction, leading to interference with the hydration of the material, which can increase the porosity and result in an unsuccessful endodontic treatment via the retrograde route ^11^.

Considering that an ideal dentin cleaning protocol for bioceramic-based materials has not been established, once studies carried out so far have yielded contradictory results ^12^, the aim of this study was to evaluate the porosity and bond strength of three types of repair materials (MTA, pure C, and C + a collagen additive [Ccol]) after blood contamination and dentin cleaning using different protocols. The null hypothesis was that the blood contamination and different cleaning protocols or compositions of the repair materials would not interfere with the material porosity or dentin bond strength.

## Materials and methods

A priori power calculation revealed that for porosity and bond strength 10 samples per subgroup had 0.96 test power, considering type α error of 5% and an effect size of 0.25. 

The attached figure, labeled as the graphical abstract, provides a summary of the study design.

### Sample selection and preparation

This study was approved by the Human Research Ethics Committee (CAAE: 14339719.2.0000.5498) and the Animals Use Ethics Committee at the University of Ribeirão Preto (CEUA 05/2019). Bovine central incisors were selected. The crowns were cross-sectioned, and a standardized root length of 17 mm was used. A line 1 mm short of the apex was demarcated with a digital caliper (Lee Tools, São Paulo, SP, Brazil), and the root apex was removed. Two disks from each root (thickness, 2 mm) were sectioned; those with a canal diameter of >2.1 and <1.8 mm were discarded, whereas those with 0.15-0.35 mm of dentin wear were included. Canal preparation was performed with a diamond-tipped cone-tip 4137 (KG Sorensen, Cotia, SP, Brazil), obtaining 195 dentin disks with a root canal diameter of approximately 2.2 mm. The samples were randomly distributed among the different groups.

White MTA (Lote 101148 - Angelus, Londrina, PR, Brazil), C, and Ccol were used in the present study (Box 1). The dentin was dried with 140 absorbent paper cones (Dentsply-Sirona, Ballaigues, Vaud, VD, Switzerland) before root canal filling. The MTA (Angelus) was handled in powdered form with distilled water at a ratio of 3:1 by volume until a sandy consistency was reached. Similarly, the C preparations were handled with distilled water at a powder/water ratio of 3:1 by volume ^3^.

**Box 1:**
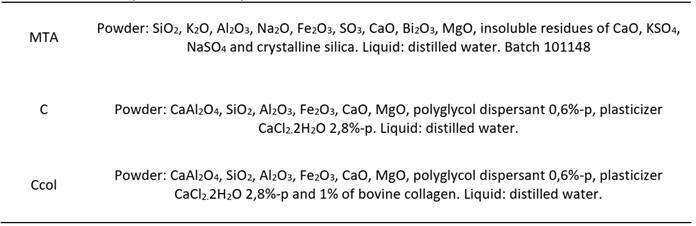
Chemical composition of the repair material used.

### Blood collection

The blood was obtained from a healthy volunteer through phlebotomy, using a 30 × 70 gauge needle (Becton Dickison, NJ, USA). In all, 50 IU of heparin (Nevparin, Mustafa Nevzat Ilaç San. A. Istanbul, Turkey) was used for each 1 mM of blood to prevent blood clotting ^13^. The blood was used immediately after collection.

### Study groups

The root dentin disks were fixed on glass slides using heated silicone glue. The samples were divided into the following groups based on the condition of the dentin during the filling process:

- N (negative control): uncontaminated dentin preparation.
- P (positive control): the tooth preparation was contaminated with 0.05 mL of blood, maintained for 5 min, following which the blood was aspirated using a Capillary Tip cannula (Ultradent, South Jordan, UT, USA) with a high-powered suction adapter.
- S (saline): the tooth preparation was contaminated with 0.05 mL of blood, kept for 5 min, and then cleaned by irrigation with 2 mL of saline (NaCl 0.9% - JP Indústria Farmacêutica S.A, Ribeirão Preto, SP, Brazil), using a disposable syringe and irrigation tip (NaviTip - Ultradent, South Jordan, UT, USA). The fluids were aspirated using a Capillary Tip (Ultradent) cannula with a high-powered suction adapter.
- Na (NaOCl 2.5%): the dentin preparation was contaminated with 0.05 mL of blood, kept for 5 min, and then cleaned by irrigation with 1 mL of 2.5% NaOCl (Asfer, São Caetano do Sul, SP, Brazil), using a disposable syringe and irrigation tip (NaviTip). The 2.5% NaOCl was allowed to act for 5 min and then aspirated using a Capillary Tip (Ultradent) cannula with a high-powered suction adapter. Then, 1 mL of saline (JP Indústria Farmacêutica S.A) was added using a disposable syringe and irrigation tip (NaviTip) and aspirated with a Capillary Tip (Ultradent) cannula and a high-powered suction adapter.
- NaE (NaOCl 2.5% + EDTA 17%): the tooth preparation was contaminated with 0.05 mL of blood, maintained for 5 min, and cleaned by irrigation with 1 mL of 2.5% NaOCl (Asfer) using a disposable syringe and irrigation tip (NaviTip). The 2.5% NaOCl (Asfer) was maintained for 5 min and aspirated with a Capillary Tip cannula. Then, 0.5 mL of 17% EDTA (Da Terra, Ribeirão Preto, SP, Brazil) was applied using a disposable syringe and irrigation tip, allowed to act for 5 min, and then aspirated with a Capillary Tip (Ultradent) cannula. Next, 0.5 mL of saline (JP Indústria Farmacêutica S.A) was applied using a disposable syringe and irrigation tip (NaviTip), followed by aspiration with a Capillary Tip (Ultradent) cannula.

The bovine root samples were further divided into three subgroups based on the type of repair material used: MTA (Angelus), C, and Ccol.

### Evaluation of the initial porosity

The samples were scanned on the SkyScan microtomograph model 1176 (Bruker micro CT, Kontich, Belgium) under the following parameters 90 kV, 278 mA, 8.6 μm isotropic resolution, 360 ° rotation around the vertical axis with 0.5 °, total quantity of 2 frames (frames), using 0.1 mm thick copper filter. The two-dimensional projections of the generated images were filed in the Tagged Image File (TIFF) format. Followed by reconstruction of the axial sections from the angular projection images using the modified Feldkamp cone beam reconstruction algorithm, using the NRecon v.1.6.9.18 program (Bruker-microCT, Kontich, Belgium). 

After reconstruction, the image was segmented using the binary or interactive threshold technique with the aid of the CTAn v.1.14.4.1 + program (Bruker microCT, Kontich, Belgium), thus obtaining a binary image where the black pixels represented the background and white pixels the object of the analysis, obtaining the volume of interest (VOI). Thus, after defining the VOI, a binarization threshold value of 130-255 was used for all C and Ccol groups, 118-255 for the NC and S MTA (Angelus) groups, 110-255 for P MTA (Angelus) and 122-255 for Na and NaE MTA (Angelus) and through the Custom processing tool, a sequence of mathematical operations (task list) was used, from which it was possible to obtain a three-dimensional quantitative analysis of the percentage of open and closed pores (%) in relation to the total volume of the repair material. In addition, three-dimensional models of each sample were also generated in the same software. In the CTVol v.2.3.2.1 program (Bruker-microCT, Kontich, Belgium), a realistic visualization of the three-dimensional models was made for the qualitative analysis of the material of each experimental group. 

### Sample storage

The samples in the N group were stored in plastic tubes with a sterile gauze (moistened in 0.5 mL of deionized water) covering the cervical and apical parts of the preparation. Similarly, the P, S, Na, and NaE samples were stored in plastic tubes. Sterile gauzes moistened in 0.5 mL of blood were used to cover the apical portions; likewise, sterile gauzes moistened in 0.5 mL of deionized water were used to cover the cervical portions of the preparations. All specimens were stored in an oven at 37°C for 28 days, and the gauzes were changed twice a week ^14^.

### Evaluation of the final porosity

After the 28-day storage period, the samples from each subgroup underwent microtomographic examinations according to the scanning and reconstruction protocols described for the initial microtomographic examination. The images of the repair material were processed and analyzed after the storage period. The difference in porosity, compared to the initial porosity values, was calculated as a percentage. Positive results represented an increase in porosity, and negative results represented a decrease.

### Evaluation of the bond strength

The Instron EMIC 23-5s universal testing machine (Instron Corporation, Canton, MA, USA) was used to perform the push-out test at a speed of 1 mm/min; a rod of 4 mm in length and 1.8-2.0 mm in diameter was used. The selected diameters allowed the stem to touch at least 70% of the reparative endodontic material surface without contacting the dentin. The force required for the displacement of the material (F) was measured in kilonewtons (kN), transformed into tension (σ), and in megapascals (MPa), using the following formula:

$$\sigma =\frac{F}{A}$$

where A, the material adhesion area, was obtained using the following formula:

A = 2 π Re h

where Re = (Rm + rm)/2, and A = π (Rm + rm) h.

Analyses of the interface and type of failure were performed using a stereoscopic magnifying glass (ZEISS, Stemi 2000-C, BV, Germany) (magnification, 25×). The failure pattern after the push-out test was classified from the images obtained as follows: a) adhesive; b) cohesive; and c) mixed.

### Evaluation of the chemical composition, morphology, and union interface

Analyses of the chemical elements, X-ray dispersive energy (EDS-X), and morphology were performed via scanning electron microscopy (SEM) using the ZEISS EVO 50 microscope (JEOL Ltd, Tokyo 190-0012, Japan) at an operating at 20 kV. Three specimens from each subgroup (n = 3) were used after storage for 28 days. The Ca/P ratio was quantified using the data obtained by the EDS-X. The representative surface and interface area were photographed at ×5000.

### Statistical analysis

The data obtained from the porosity and push-out tests were subjected to preliminary statistical tests to verify the sample distribution. After confirming the homogeneity (Levene's test) and normality (Shapiro-Wilk's test), two-way Analysis of Variance (ANOVA) (α = 0.05) and Tukey's post hoc tests were used to determine the differences among groups (α = 0.001). Statistical analysis was performed using the SigmaStat 3.5 software (Systat Software; San Jose, CA, USA). Additionally, the data obtained by EDS-X, the SEM images, and the failure patterns were qualitatively analyzed.

## Results

### Porosity

The ANOVA test showed statistically significant differences in porosity among the three types of materials; likewise, significant differences were observed among samples based on the dentin condition (p <0.001). An interaction between the factors type of material and cleaning protocol was observed (p <0.001). The Tukey test revealed that the porosity values of samples sealed with MTA were lower than those sealed with C (p = 0.02) and higher than those with the Ccol additive (p <0.001). When comparing the dentinal condition, contact with blood resulted in significantly higher porosity values than those observed in samples without contamination (p <0.001), regardless of the cleaning and sealer protocol used. 

**Figure 1:**
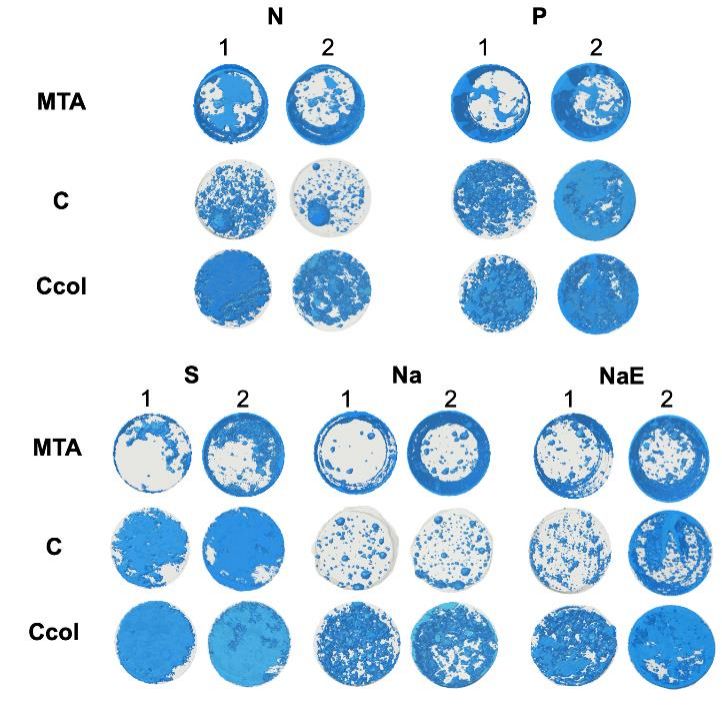
Aspect of the porosity of the samples analyzed by microcomputed tomography. 1 indicates samples before storage and 2 after storage. “N” is negative control; “P” is positive control; “S” is saline; “Na” is Sodium hypochlorite; “NaE” is Sodium hypochlorite + EDTA.

Table 1 presents the average values and standard deviations of the variations in porosity in the different groups (cleaning protocols) and subgroups studied (sealer type). Figure 1 shows the images obtained for analysis via microcomputed tomography (CT); except for N, the sealers showed increased porosity after 28 days of storage in the other groups.

**Table 1:** Porosity (%) mean values and standard deviation of the repair material according to the different dentin condition.

Dentin condition	Repair material		
	MTA	C	Ccol
N	-0.51±0.76^C^_a_	-2.95±1.50^D^_b_	-12.77±2.30^C^_c_
P	3.87±1.03^AB^_b_	7.74±1.56^A^_a_	5.05±1.76^A^_b_
S	5.33±2.25^A^_a_	3.56±1.94^B^_b_	2.60±1.46^B^_b_
Na	2.75±0.81^B^_ab_	1.31±0.60^C^_b_	3.09±1.29^B^_a_
NaE	3.34±1.35^B^_b_	9.27±1.35^A^_a_	4.29±1.70^AB^_b_

Same lowercase letter indicate statistical similarity with the data at the same row

Same uppercase letter indicate statistical similarity with the data at the same column

### Bond strength

Statistically significant differences in bond strength were observed based on the type of sealer used and the condition of the dentin (p <0.001). There was no interaction between these factors (p = 0.887). The Tukey test revealed that the values in the MTA-treated samples were similar to those in the Ccol-treated samples (p = 0.777). The values in the C subgroup were significantly higher than those in the MTA subgroup (p = 0.028) but similar to those in the Ccol subgroup (p = 0.142).

When comparing the dentinal condition, blood contamination without any cleaning protocol significantly lowered the bond strength when compared to that in samples that were not contaminated (p <0.001). The bond strengths of samples in the S and Na groups were similar (p = 0.969); additionally, the values were greater than that in the P group and lower than that in the N group (p <0.001). The NaE group presented with values similar to that in the P group (p = 0.995) and lower than those in the other groups (p <0.001). Table 2 shows the mean bond strength values ± standard deviation based on the dentinal condition.

**Figure 2:**
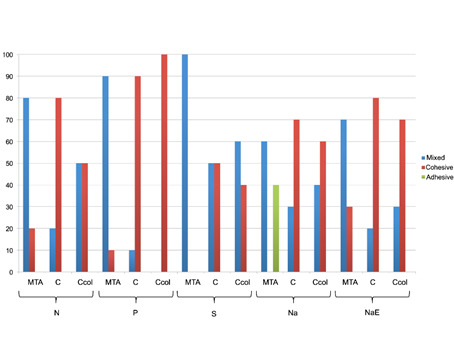
Type of failure after push-out test of all groups/subgroups.

Analysis of the type of failure showed a predominance of mixed and cohesive failures (≥50%) for all the tested sealers, regardless of the dentinal condition (Figure 2). Adhesive failures were seen only in MTA-Na subgroup.

### Chemical composition, morphology, and bond interface

**Figure 3:**
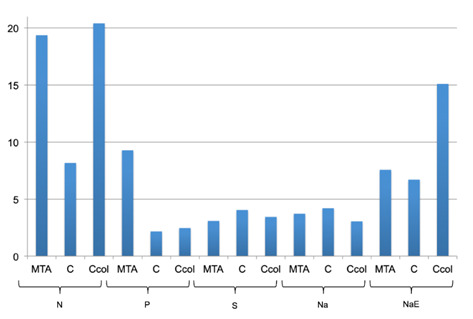
Ca/P ratio means values of the samples surface of all groups/subgroups.

**Figure 4:**
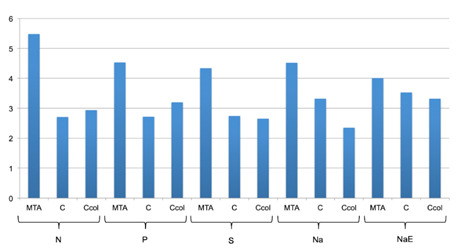
Ca/P ratio means values of the samples interface of all groups/subgroups.

**Figure 5:**
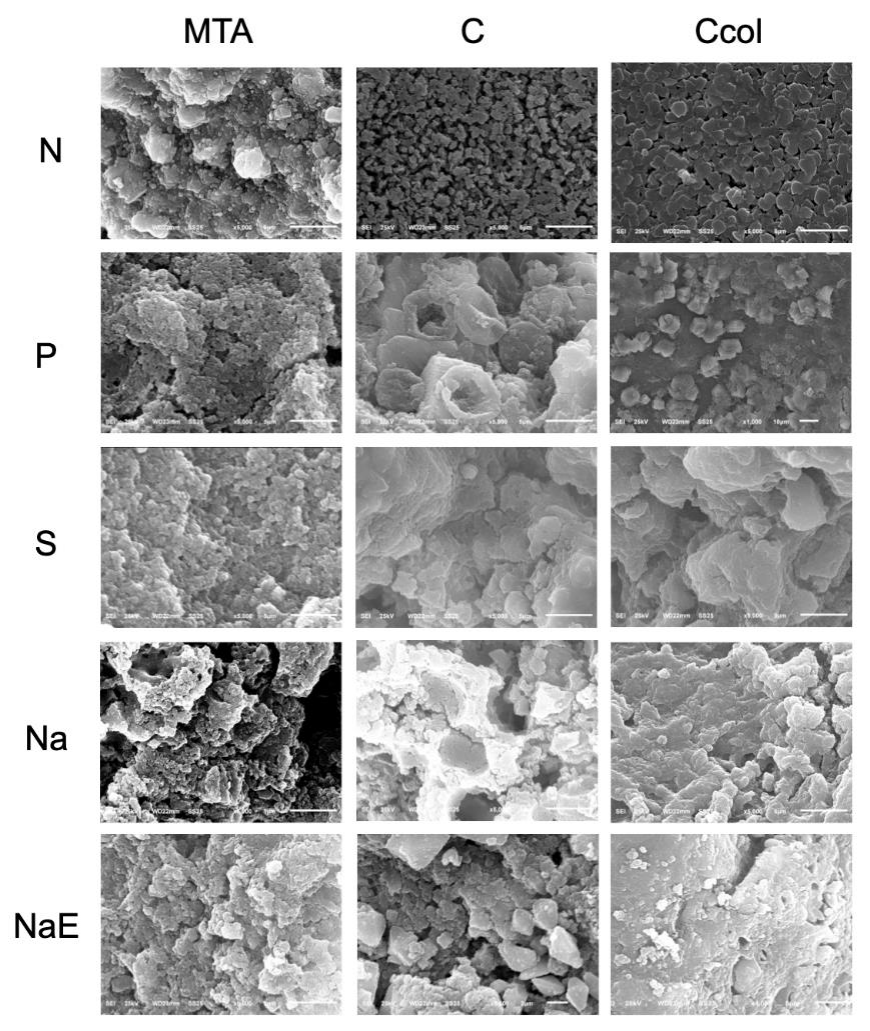
Scanning electron microscopy images at ×5,000 of the sample surface of all groups/subgroups.

**Figure 6:**
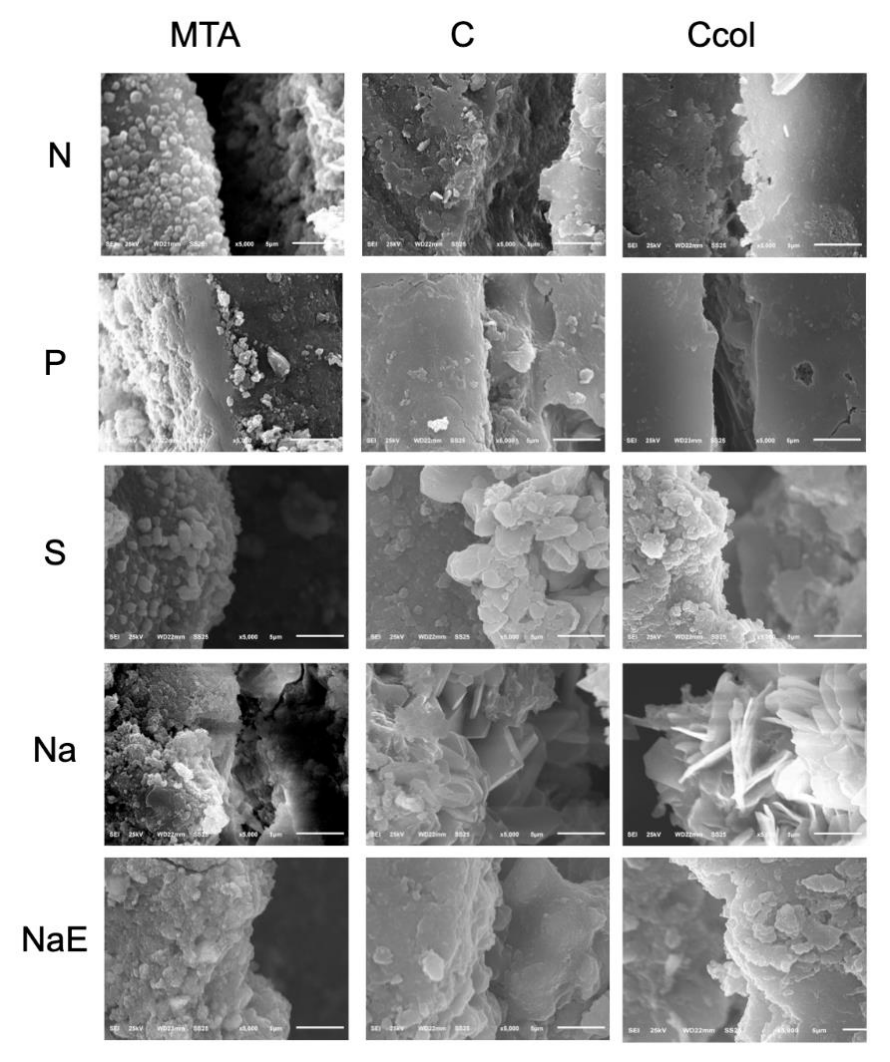
Scanning electron microscopy images at ×5,000 of the samples interface of all groups/subgroups.

The chemical composition of the sealer surfaces and the dentin/sealer interface are shown in Figures 3 and 4, respectively; Ca²^+^ and PO_4_^3−^ were identified in all the samples analyzed. None of the samples, both on the surface and at the interface, showed Ca/P ratio values (Figures 3 and 4) similar to human hydroxyapatite (1.5-1.7) ^20^. In the images obtained by SEM Figures 5 and 6, the samples in the N group had a regular surface compared to those in the other groups. No crystalline precipitates were observed on the analyzed sealers, regardless of the dentinal condition. In the interface evaluation, only the Na samples showed acicular structures, similar to crystals, on the dentin.

**Table 2:** Bond strength mean values and standard deviation according to dentin condition and repair material.

Dentin condition	Repair material			Total mean
	MTA	C	Ccol	
N	4.15±0.28	4.59±0.56	4.24±0.81	4.33±0.65^A^
P	2.27±0.30	2.71±0.56	2.36±0.75	2.45±0.58^C^
S	3.06±0.29	3.11±0.46	3.00±0.33	3.06±0.36^B^
Na	3.12±0.53	3.09±0.26	3.31±0.82	3.15±0.57^B^
NaE	2.34±0.38	2.54±0.40	2.41±0.37	2.39±0.41^C^
Total mean	32.96±0.80^A^	3.23±0.87^B^	3.03±0.92^AB^	

Same uppercase letter indicate statistical similarity with the data at the same column or row

## Discussion

The success of parendodontic surgery is directly related to the sealing and stability of the sealer used to fill the dentin cavity. Considering the possibility of blood contamination of the sealer during the surgical procedure ^11^ ^,^ ^14^, it is essential to evaluate the effectiveness of different cleaning protocols and to assess their effects on the porosity, sealing quality, and resistance to displacement of retrofilling materials at the dental apex. The null hypothesis was rejected in the present study because differences in porosity and bond strength were observed between the calcium aluminate and calcium silicate sealers. In addition, significant differences in porosity and bond strength were observed among the three types of sealers used and among the various types of dentinal conditions.

High-resolution micro-CT analysis is used to assess the dimensional changes and porosity of endodontic materials using high-precision three-dimensional quantitative data ^15^. Owing to the nondestructive nature of this method, the internal structure and the porosity of the material can be determined from the images at different experimental time points ^16^. Therefore, in the present study, we used this technique to assess the porosity of the bioceramic sealers before and after contamination with blood and using different dentin cleaning protocols.

In addition to the porosity and sealing capacity, the result of the reaction between the material and the dental substrate was assessed. Bioceramic materials are considered bioactive due to their ability to form compounds derived from apatite, which increases their retention in the dentinal cavity ^2^ and favors the adhesion and spreading of osteoprogenitor cells ^4^. SEM and EDS-X were used to evaluate the morphology and identify the chemical elements formed on the sealers and at the interface with the root dentin.

In the analysis of porosity for the different sealers, there is a lack of results for calcium aluminate cements; however, alterations in this property owing to storage in physiological solutions have been reported in the case of the calcium silicate sealers as MTA. Previous studies have reported a reduction in the porosity of MTA after immersion in phosphate-buffered saline (PBS) ^17^. As a storage medium, PBS can favor the formation of hydroxyapatite crystals ^2^ and improve the distribution of sealer particles ^17^, thereby reducing its porosity. However, in the current study, the samples were stored in deionized water or blood, resulting in increased porosity of the contaminated samples; this outcome may be related to alterations in the crystalline phases of the materials during hydration and to the changes that occur during the hardening process ^13^. During hydration, the MTA presents as an amorphous calcium silicate hydrate gel, which adds to the formation of calcium hydroxide, contributes to the precipitation of hydroxyapatite crystals, and decreases the porosity of the material ^18^. Exposure to blood proteins inhibits the formation of calcium hydroxide and the hydration process, thus decreasing hydroxyapatite formation ^18^. This finding was corroborated to the Ca/P results as hydroxyapatite was not identified at the interface of sealers in contact with blood. Furthermore, the increase in porosity may be related to the leaching of components from the material, mainly during the hydration process when the mechanical resistance of the material is significantly low ^18^. In the current study, this hypothesis was corroborated by the presence of a regular surface on the uncontaminated samples compared to those on samples contaminated with blood.

The reactions in samples containing the C formulations were probably similar to those containing MTA because the porosity of the contaminated samples was significantly higher than that in samples without contamination. Samples with collagen additive showed a significantly lower porosity than those with pure C, which may be related to the more intense bioactivity reaction; a greater release of calcium during the hydration reaction has been observed in the study by Oliveira et al. ^7^.

The dentin cleaning protocols influenced the porosity of the materials in the current study. EDTA chelating solution resulted in the highest porosity values, which were similar to those seen in the positive controls (samples contaminated with blood without any cleaning protocol). These results can be justified by the demineralization of the chelating solutions due to the removal of calcium from the root dentin, thus interfering with hydroxyapatite formation ^12^. However, less porosity was observed when only NaOCl was used. In the present study, crystals similar to hydroxyapatite were observed only in the NaOCl group. The decomposition of blood proteins by NaOCl ^19^ may have favored the reactions that occur during hydroxyapatite formation. Nevertheless, the obtained results are related to an in vitro evaluation, and despite significant control of the involved variables, they may not accurately simulate clinical conditions. In this regard, the constant renewal of body fluids could impact favorably on results for both the use of EDTA and NaOCl.

The push-out test was performed because it allows for evaluating the displacement resistance of the material inserted in the root canal. This test is widely used for its effectiveness and reliability (2, 14). Bioceramic materials are subject to the forces of fluids from the periradicular region or to the interposition of biomaterials used for the formation of new bone. Thus, it is important to perform the push-out test to simulate the displacement of the sealer from its cavity ^2^ ^,^ ^20^.

Considering the comparison of bond strength between calcium silicate and calcium aluminate sealers, Do Carmo et al. ^2^ reported similar bond strengths in the MTA and C sealers, whereas, in the present study, C presented with higher bond strength than MTA. The results observed in the study by Do Carmo et al. ^2^ may be related to differences in the composition of C because the authors used bismuth oxide as a radiopacifier. Bismuth oxide can interfere with the physicochemical properties and the hydroxyapatite crystal-forming capabilities of the MTA sealer ^11^. Similarly, bismuth oxide may have altered the flow and hydroxyapatite formation abilities of the C sealer, which could account for the lower bond strength observed in the study by Do Carmo et al. ^2^. No differences in bond strength were observed between the Ccol samples and the C- and MTA-treated samples in the present study. The formulation of C + the collagen additive is unprecedented in the scientific literature, which limits the comparison of the results obtained in the current study.

A systematic literature review and meta-analysis noted conflicting data regarding the effect of blood on calcium silicate-based reparative materials ^10^. However, there is strong evidence that blood may compromise the interaction of these materials with dentin walls, thereby reducing the bond strength of these materials ^10^. Corroborating with these results, in the present study, the samples in contact with blood showed significantly lower bond strength values than the samples without contact, regardless of the type of reparative material evaluated. These results may be related to the interference of blood in the cement hydration process or with the inhibition of hydroxyapatite maturation at the dentin/material interface as blood proteins can interfere with the transformation of amorphous calcium phosphate into carbonated apatite ^21^ ^,^ ^22^. Nonetheless, as observed in the present study, the increase in the porosity of the samples contaminated with blood may have reduced the contact of the sealers with the dentinal walls. Previous studies have shown that mineralization can increase the resistance against displacement forces in the dentin cavity for both MTA ^23^ and C ^2^. The low bond strength values in samples contaminated with blood in the current study are in accordance with the findings of previous studies on calcium silicate-based sealers ^14^, thus justifying the need for cleaning and backwash treatment protocols prior to the insertion of calcium aluminate-based sealers.

The use of EDTA resulted in the lowest bond strength values, similar to that in the positive control and lower than that in the negative control. The use of chelating agents can interrupt the hydration of the material by chelating the calcium ions, altering the Ca/P ratio, and directly interfering with the chemical adhesion of the sealer to the dentin ^24^. On the other hand, the saline- and NaOCl-treated groups showed values that were higher than those in the EDTA-treated and positive control samples. Similar to the porosity results, the increased bond strength in the NaOCl-treated samples may be attributed to the degradation of the blood protein components and the elevation of the pH, which favored the chemical bonding of the sealer. However, NaOCl can cause proteolytic changes in the root dentin ^25^; therefore, the results were not similar to those observed in the negative controls.

These results have significant clinical implications as they provide evidence for choosing an irrigating solution that could minimize the effects of blood on the interaction of the restorative material with dentin. In this regard, physiological saline or 2.5% NaOCl could be used before the insertion of reparative materials in areas of dentin previously contaminated with blood. However, although the use of NaOCl and saline led to better results than those in the positive control, none of the protocols completely eliminated the effects of blood on the porosity and bond strength of the bioceramic sealers. Therefore, additional studies are necessary to develop cleaning protocols that can neutralize the effects of blood on root dentin without changing its composition and impairing the reactions with bioceramic sealers. With regard to the addition of collagen, better results were observed only in terms of the porosity when compared to the original formulation in the C samples. Additional studies using different methodologies are required to assess the physical-chemical and biological properties of new materials.

Overall it can be concluded that the contamination with blood increased the porosity of the calcium silicate- and calcium aluminate-based sealers, but dentin cleaning with 2.5% NaOCl minimized the effect on the evaluated parameters. The collagen additive decreased the porosity compared to that seen in samples with the pure calcium aluminate formulation. Blood contamination reduced the bond strengths of the calcium silicate- and calcium aluminate-based sealers, and cleaning with saline or 2.5% NaOCl reduced this effect.
